# Comparison of Remotely Applied and Face-to-Face Disease Activity Scores in Saudi Arabian Patients With Rheumatoid Arthritis: A Prospective Cohort Study

**DOI:** 10.7759/cureus.52390

**Published:** 2024-01-16

**Authors:** Abdulrahman Y Almansouri, Rahaf Alsofyani, Hanin A Alharbi, Ahmed S Almaqati, Hind S Aloqbi, Lama Bakhsh, Alaa Althubaiti, Zeyad Alzahrani

**Affiliations:** 1 Department of Medicine, King Abdulaziz Medical City, Jeddah, SAU; 2 Department of Medicine, King Faisal Specialist Hospital and Research Center, Madinah, SAU; 3 Internal Medicine, King Abdullah International Medical Research Center, Jeddah, SAU; 4 Department of Medicine/Rheumatology, King Abdulaziz Medical City, Jeddah, SAU; 5 Department of Medicine, King Fahad Hospital, Tabuk, SAU; 6 College of Medicine, King Saud Bin Abdulaziz University for Health and Sciences, Jeddah, SAU

**Keywords:** covid-19, telemedicine, saudi arabia, patients satisfaction, disease activity in rheumatoid arthritis

## Abstract

Objectives: This study aimed to assess the disease activity indices (DAI) of rheumatoid arthritis (RA) by telephone-based tele-visits compared to face-to-face clinic encounters.

Methods: Patients with RA attending outpatient clinics between December 2021 and May 2022 were prospectively recruited. Disease activity assessments were initially performed in the clinic using the disease activity score 28-C-reactive protein (DAS28-CRP) and disease activity score 28-erythrocyte sedimentation rate (DAS28-ESR). Within two weeks of the clinic visit, a telephone-based assessment gathered information on demographics, Routine Assessment of Patient Index Data 3 (RAPID3) score, and satisfaction. Disease activity scores were dichotomized into remission or low disease activity and moderate to high disease activity.

Results: A total of 78 patients completed the two-point interview. Of those, 62 (79.49%) were women, with a mean age of 54.73±13.71 years. Seropositivity for rheumatoid factor and/or anti-citrullinated peptide was observed in 51 (83.61%) participants. Twenty-seven percent of the patients were classified as in remission or low disease activity by RAPID3. This was 71% for DAS28-CRP and 33% for DAS28-ESR. Based on the dichotomized disease activity classification, the agreement percentage between RAPID3 and DAS28-ESR was 78.08%, while it was 47.22% between RAPID3 and DAS28-CRP, which resulted in kappa statistic values of 0.48 (moderate agreement) and 0.14 (low agreement), respectively. Satisfaction rates were low.

Conclusion: Telephone-based RAPID3 showed a low-moderate agreeability compared to DAS28 and had low satisfaction rates. This suggests that tele-rheumatology care by this means was not feasible for following up with patients with RA and warrants further development.

## Introduction

Prior to the emergence of the coronavirus disease 2019 (COVID-19) pandemic, telemedicine was used to provide care for remote areas in developed nations and trans-continental specialized consultation. After the pandemic emerged, global and national demand for telemedicine grew to reduce the need for physical contact and prevent the spread of infection [1]. As a response to the situation in our hospital, telemedicine rheumatology clinics that were telephone-based have been implemented. However, their efficacy was unknown.

Disease activity score 28-C-reactive protein/erythrocyte sedimentation rate (DAS28-CRP/ESR) and Routine Assessment of Patient Index Data 3 (RAPID3) are well-known disease activity indices (DAI) that have been used to assess rheumatoid arthritis (RA) disease activity [2-5]. These scoring systems indicate whether the patient is in remission or has a low, moderate, or high disease activity, which is essential for implementing a treat-to-target (TTT) concept. The current TTT in RA aims to achieve remission or a low disease activity status [6,7]. Furthermore, some DAI have been used in telemedicine, such as the RAPID3 score [3,8-10].

To the best of our knowledge, this is the first study to assess telemedicine care in rheumatology practice in Saudi Arabia. Our primary objective was to assess the efficacy of telemedicine practice implemented during the COVID-19 pandemic. We estimated disease activity in patients with RA remotely and compared the results with those applied in the clinic. As a secondary objective, satisfaction with telemedicine was assessed through an approved questionnaire.

## Materials and methods

Study design

Patients with RA who visited our outpatient ambulatory department (OPD) at King Abdulaziz Medical City, Jeddah, Saudi Arabia, between December 2021 and May 2022 and fulfilled the selection criteria (indicated below) were recruited. Participants were interviewed in person in the OPD clinics, and informed consent was obtained. All interviews were conducted in Arabic language. The DAS28 survey was administered by consultants or adult rheumatology fellows affiliated with our institute. To complete the two-point assessment, patients were interviewed remotely via telephone by applying the RAPID3 score assessment. Additionally, demographics and an inquiry on satisfaction with the tele-visit were obtained. The inquiry on satisfaction was composed of three questions. The first two were yes/no questions: 1) If the patient was satisfied with tele-visit; 2) If the patient would like to continue tele-visits. The last question was on how they felt about the efficacy of tele-visit in comparison to face-to-face (F2F); was it less, as or more effective than the usual F2F visits? This interview was conducted by three senior medical residents affiliated with our hospital and took place within two weeks of the F2F.

Inclusion and exclusion criteria

We included adult patients (aged 18 years and above) with a diagnosis of RA who accepted an invitation to participate in our project, signed informed consent forms, and completed a clinic-based F2F assessment of their disease activity followed by the telephone-based RAPID3 survey within two weeks. We included patients who had either ESR and/or CRP performed recently to be able to apply either DAS28 CRP/ESR or both. The exclusion criteria were newly diagnosed RA, refusal to participate in the study, and/or presence of an overlapping connective tissue disease.

Disease activity index

The RAPID3 is composed of functional assessments found in the multidimensional HAQ (MDHAQ) questionnaire, which comprises one-third of the total score, while the other two-thirds are equally distributed between patient-reported pain and overall functioning [3]. The patient’s total score, out of 30, was used to classify disease activity as either in remission (<3), low (3.1-6), moderate (6.1-12), or high disease activity (>12). Because of its nature (i.e., composed of a set of questions that can be asked on the telephone without the need for physical examination), it has been chosen for telephonic use. The authors of the RAPID3 score provided an Arabic-validated form of the MDHAQ questionnaire that has been used over the telephone for RAPID3 scoring and demographic data collection.

The DAS28 results in a similar DAI classification; however, it requires a clinician’s joint count assessment for tenderness and swelling and laboratory measurements of CRP or ESR. After applying the formula, DAS28-ESR remission (less than 2.6), low (2.6-3.2), moderate (3.3-5.1), or high (>5.1) and DAS28-CRP remission (less than 2.6), low (2.6-3.2), moderate (3.3-4.6), or high (>4.6) disease activity was also indicated [11].

Ethical consideration and copy-rights licensing

Informed consent was obtained from all participants. The participants’ privacy and confidentiality were assured, no identifiers were collected, and all hard and soft copies of data were stored in a secure and safe location within our institute’s premises and could only be accessed by the research team. The study was approved by the local Institutional Review Board (IRB) office on November 10, 2021, under protocol number NRJ21J/102/04. This study was conducted in accordance with the principles of the Declaration of Helsinki. A license agreement from the copyright author of the RAPID3 score, Dr. Theodore Pincus, was obtained for our study, in agreement with RWS Life Sciences [3].

Statistical analysis

All statistical analyses were performed using JMP version 8.1 (SAS Institute Inc., Cary, NC, USA). Categorical variables are described as frequencies and percentages. For numerical variables, the normality of the distribution was assessed using the Shapiro-Wilk test. Variables following normal distribution are presented using mean and standard deviation, and variables not normally distributed are described using median (range: minimum-maximum). Missing data were included in the analyses as their own without imputation. Disease activity was classified as remission, low, moderate, or high. Additionally, disease activity classification was dichotomized as planned before data collection into remission and low disease activity and moderate and high disease activity for better correlation and implications of the TTT strategy in the analysis. As the RAPID3 is the reference scale for comparisons across different scales, the agreement between the RAPID3 and other scores was evaluated using different approaches. The level of agreement was evaluated using Cohen’s kappa statistic [12]. For graphical analysis, a Bland-Altman plot was used to assess the scores for bias by plotting the difference between the RAPID3 scale and DAS28 against the mean of the two DAS28 scales [13]. There were no a priori acceptable limits of agreement; therefore, a 95% confidence interval was used as the limit of agreement. For better interpretation, the DAS28-CRP and DAS28-ESR were rescaled using a constant factor to compare these measures with those of RAPID3 (range: zero to 30).

We used additional statistical tests to explore the differences in satisfaction with the teleclinic between two patient groups: those with low-to-remission disease activities and those with moderate-to-high disease activities. Additionally, we aimed to investigate the relationship between patient characteristics and satisfaction with tele-visits. To achieve this, we implemented the independent sample t-test, Mann-Whitney U test, Pearson’s chi-squared test, or Fisher’s exact test. Due to this study’s exploratory nature, no adjustment for multiple testing was performed; thus, these results should be interpreted with caution due to the risk of an inflated type I error. A p-value of <0.05 was considered statistically significant.

A minimum sample size was determined based on a pilot analysis and knowledge of the statistical measure to be used [14]. Assuming an adequate precision of ±0.4 times the standard deviations, a sample size of 80 paired measurements was estimated to obtain, a 95% confidence interval for the Bland-Altman 95% limits of agreement. However, 78 patients met the inclusion and exclusion criteria.

## Results

Patient characteristics

Table 1 shows the patient characteristics and missing data. The mean ± standard deviation of the patient's age was 54.73±13.71 (range: 24-82) years, with 79.49% women. The median disease duration was nine years (min-max: 0.5-44) years. The median years of education is nine years, with a range of zero to 22 years.

**Table 1:** Patients baseline characteristics BMI, body mass index; bDMARD, biologic disease-modifying anti-rheumatic drugs (included adalimumab, etanercept, or tocilizumab); csDMARD, conventional synthetic disease-modifying anti-rheumatic drugs (including methotrexate and/or hydroxychloroquine or leflunomide); tsDMARDS, targeted synthetic disease-modifying anti-rheumatic drugs (including upadacitinib or tofacitinib); RAPID3, Routine Assessment of Patient Index Data 3; DAS28, disease activity score 28; CRP, C-reactive protein; ESR, erythrocyte sedimentation rate; SD, standard deviation; n, number of observations

Total patients	78
Variables	Descriptive statistics
Age, mean ± SD years	54.73±13.71
Female sex, n (%)	62 (79.49)
BMI, mean ± SD cm	30.69±6.22
Education, median (min-max) years	9 (0-22)
(n missing = 1)	-
Smoking, n (%)	7 (9.09)
(n missing = 1)	-
Employment, n (%)	-
Unemployed	52 (68.42)
Employed	10 (13.16)
Retired	14 (18.42)
(n missing = 2)	-
Marital status, n (%)	-
Married	56 (76.71)
Widow	13 (17.81)
Divorced or single	4 (5.48)
(n missing = 5)	-
Comorbidity, n(%)	58 (74.36)
Time from diagnosis of rheumatoid arthritis, median (range: min-max) years	9 (0.5-44)
(n missing = 19)	-
Seropositivity, n (%)	-
Positive	51 (83.61)
Negative	10 (16.39)
(n missing = 17)	-
Fibromyalgia, n (%)	4 (5.19)
(n missing = 1)	-
Type of treatment, n (%)	-
bDMARD	29 (38.16)
csDMARD	44 (57.89)
tsDMARD	3 (3.95)
(n missing = 2)	-
RAPID3 and DAS28 disease activity index, mean ± SD (range: min-max)	-
RAPID3	11.40±7.85 (0-28)
DAS28-CRP	2.77± 1.07 (0.72-5.69)
(n missing = 6)	-
DAS28-ESR	3.63±1.02 (0.97-6.18)
(n missing = 5)	-

Agreement of the scores between tele-visit disease activity scale versus F2F scores

**Figure 1:**
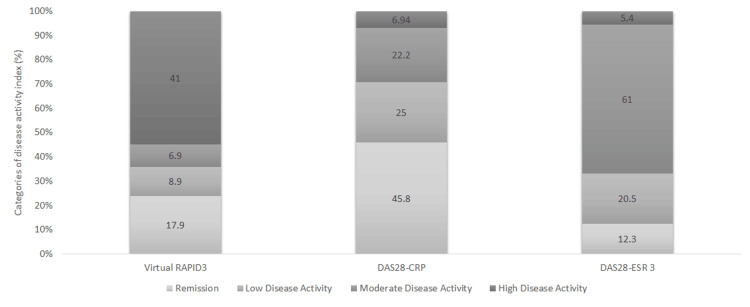
RAPID3, DAS28-CRP, and DAS28-ESR percentages of different categories of DAI DAI, disease activity indices; DAS28-CRP, disease activity score 28-C-reactive protein; DAS28-ESR, disease activity score 28-erythrocyte sedimentation rate; RAPID3, Routine Assessment of Patient Index Data 3

Figure 1 shows the percentages of the four DAI categories. Agreement percentages between RAPID3 and DAS28 DAI are shown in Table 2 and were the highest for RAPID3/DAS28-ESR (78.08%). Based on the dichotomized disease activity scores, DAS28-CRP and RAPID3 recorded an agreement of 26.39% and 20.83% in the moderate or high activity category and the remission or low activity category, respectively, with a kappa statistic value of 0.14, indicating a low agreement. In contrast, DAS28-ESR and RAPID3 recorded an agreement of 58.90% in the moderate or high activity category and 19.18% in the remission or low activity category, with a kappa statistic value of 0.48, indicating moderate agreement.

**Table 2:** Agreement percentages between RAPID3, DAS28-CRP, and DAS28-ESR DAS28-CRP, disease activity score 28-C-reactive protein; DAS28-ESR, disease activity score 28-erythrocyte sedimentation rate; RAPID3, Routine Assessment of Patient Index Data 3

Agreement of RAPID3/DAS28-CRP	Total	Remission or low disease activity	Moderate or high disease activity
Agreement, n (%)	34 (47.22)	15 (20.83)	19 (26.39)
No agreement, n (%)	38 (52.78)	N/A	N/A
n missing for DAS28-CRP = 6	-	-	-
Agreement of RAPID3/DAS28-ESR	Total	Remission or low disease activity	Moderate or high disease activity
Agreement, n (%)	57 (78.08)	14 (19.18)	43 (58.90)
No agreement, n (%)	16 (21.92)	N/A	N/A
n missing for DAS28-ESR = 5	-	-	-

**Figure 2:**
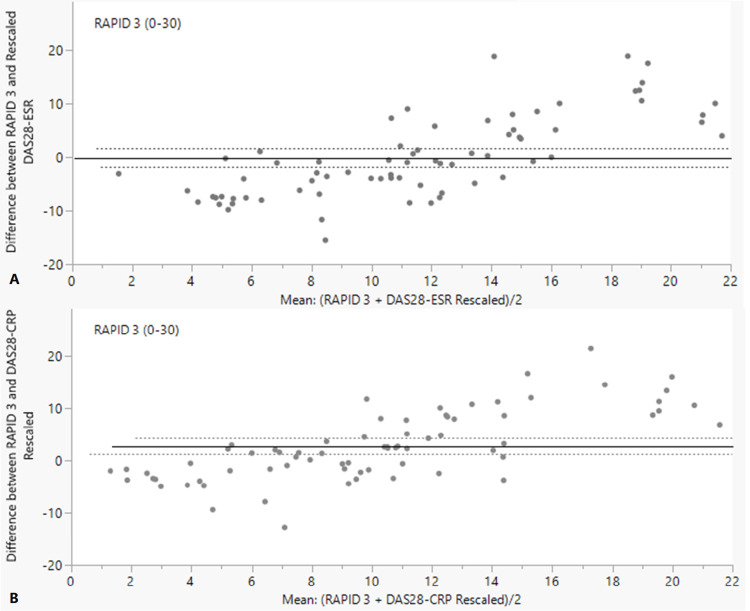
Bland-Altman plot of RAPID3 and rescaled DAS28-ESR (A) and DAS28-CRP (B) A) Horizontal lines are shown to indicate the mean difference and 95% limit of agreement (mean difference ± 1.96 × standard deviation). Good agreement is represented by a small mean difference (nearer to zero) and low dispersion around the mean difference (i.e., most points are within the dotted lines). B) Bland-Altman plot of RAPID3 and rescaled DAS28-CRP. Horizontal lines are shown to indicate the mean difference and 95% limit of agreement (mean difference ± 1.96 × standard deviation). Good agreement is represented by a small mean difference (nearer to zero) and low dispersion around the mean difference (i.e., most points are within the dotted lines).

The Bland-Altman plots (Figure 2) indicate a trend of points falling below the 95% confidence limit for low disease activity and above the 95% confidence limit for severe disease activity. In Figure 2A, the mean difference was -0.126 (close to zero); however, most data points did not fall within the 95% confidence limit of agreement. Similar results are shown in Figure 2B, and a significant mean difference of 2.7 (p-value = 0.0004) was found, indicating a systematic bias in which RAPID3 overestimated disease activity compared to DAS28-CRP. The Bland-Altman plots indicate that DAS28 did not have a strong agreement with RAPID3.

Satisfaction with tele-visit

As shown in Table 3, approximately half of our patients were not satisfied with the tele-visit. When asked, approximately 59% indicated that they would not like it to continue, as they felt it was less effective. This also remained true for both patients with low-to-remission (the number of patients who did not desire to continue tele-visiting was 12 (57.17%) out of 21 patients) and moderate-to-high disease activity (around 34 (60.71%) out of 56 patients did not desire to continue tele-visiting). The difference was not statistically significant when Pearson’s chi-square test was performed (p = 0.77).

**Table 3:** Elements of satisfaction with tele-visit F2F, face-to-face

Total patients	77
Variable	Descriptive statistics
If the patient was satisfied with the tele-visit? n (%)	
Yes	36 (46.75)
No	41 (53.25)
If the patient would like to continue tele-visit? n (%)	
Yes	31 (40.26)
No	46 (59.74)
Efficacy of tele-visit in comparison to F2F? n (%)	
As effective	20 (25.97)
More effective	8 (10.39)
Less effective	49 (63.64)

The association between satisfaction with the tele-visits and patient characteristics was examined. The results showed a statistically significant association between satisfaction and gender. Women reported increased satisfaction with tele-visits compared to men (52.46% vs. 25%, p = 0.044). Regarding the patients’ years of education, the results of the Mann-Whitney U test showed that patients who were not satisfied with the tele-visit had more years of education (median 12, range: zero to 22 years) compared to patients who were satisfied with the tele-visit (median six, range: zero to 20 years); however, the difference was not statistically significant (p = 0.052). No other differences in satisfaction according to patient characteristics (age, employment status, marital status, or time from diagnosis) were noted (p > 0.05).

## Discussion

Telemedicine visits have replaced regular in-person F2F visits at our hospital during the COVID-19 pandemic. These were largely telephone-based visits for new, follow-up, and other non-life-threatening urgent cases. The efficacy of telemedicine care at our hospital needs to be assessed in current and future practices. We selected patients with RA because it is among the most common diseases dealt with in our practice. Additionally, we used the RAPID3 survey as it is a validated tool that has been used to assess RA disease activity for follow-up patients via telephone [1,3,10,15]. Based on the dichotomized disease activity classification, the agreement percentage between the RAPID3 and DAS28-ESR was 78.08%, while it was 47.22% between the RAPID3 and DAS28-CRP, which resulted in Kappa statistic values of 0.48 (moderate agreement) and 0.14 (low agreement), respectively. Bland-Altman plots comparing the RAPID3 scores with those of the DAS28-ESR and DAS28-CRP revealed a mean difference of -0.126 (close to zero) with the former and of 2.7 with the latter (p = 0.0004), indicating poor agreement.

The combination of these findings suggests that telephone-based disease activity monitoring for RA patients follow-up had a poor agreement with conventional F2F clinic visits. This implies that telemedicine clinics for disease activity monitoring may not be practical in the current setting thus requiring further development. Of interest, these results might be related to the utility of the RAPID3 score and are contrary to recently published guidelines [15-17].

Although several studies have reported patient satisfaction with telemedicine care in rheumatology, our cohort is at odds with this finding [1,17-21]. Approximately 41 (53.25%) patients reported that they were not satisfied with tele-visits because they felt it was less effective. When exploring related factors, this did not appear to be related to technological literacy, as observed in other studies [20]. In fact, those with more education were less satisfied; however, this difference was not statistically significant. Furthermore, women appeared to be more satisfied with their tele-visits than men, which was also recently reported in another study [21]. Finally, we did not find any correlation with the disease activity severity.

This study has a few limitations, which include being conducted in a single center; thus, a multi-center large-scale study can be beneficial in terms of having a more representative sample of the Saudi population that can be generalized to other clinical practices. In addition, we assessed patients over the phone within two weeks of their clinic visit, a period that is somewhat prolonged with a possibility of DAI to change. We did not aim to assess patient/provider satisfaction in this study as a primary outcome; however, this area requires further exploration in future studies. Several measures need to be further assessed including patient satisfaction and education that were previously shown to have an impact [21-22]. During the post-pandemic era, telemedicine video-based clinics were established at our hospital. This can be an additional advantage as shown in other studies [8,10]. However, these video-based clinics require further assessment in our population.

## Conclusions

Telephone-based RA assessment had a low-moderate agreeability compared to F2F visits. This suggests that the integration of telephone-based clinics into our healthcare practices for the purpose of RA patient follow-up may not be feasible and warrants further development. Video-based clinics that were established recently need further evaluation. 
